# Body Mass Index Trends before and during the COVID-19 Pandemic in Primary School Students in Split-Dalmatia County, Croatia: A Retrospective Study

**DOI:** 10.3390/nu16010050

**Published:** 2023-12-22

**Authors:** Luka Androja, Tonči Bavčević, Anamarija Jurčev Savičević, Damir Bavčević, Jasna Ninčević, Anita Buljan, Diana Nonković, Vitor Rodrigues, Željka Karin

**Affiliations:** 1 School of Medicine, University of Split, 21000 Split, Croatia; luka.androja@aspira.hr (L.A.);; 2 Department of Sports Management, Aspira University of Applied Sciences, 21000 Split, Croatia; 3 Faculty of Kinesiology, University of Split, 21000 Split, Croatia; 4 Teaching Institute for Public Health of Split-Dalmatia County, 21000 Split, Croatia; 5 Department of Health Studies, University of Split, 21000 Split, Croatia; 6 Faculty of Medicine, University of Coimbra, 3030-222 Coimbra, Portugal

**Keywords:** body mass index, overweight, obesity, COVID-19 pandemic, health, children

## Abstract

Within the last decade, childhood obesity has become a serious problem, especially during the COVID-19 pandemic. This research paper aimed to examine whether body mass index (BMI) was higher during the pandemic (2020–2022) than in the pre-pandemic period (2012/2013–2019) using trends related to sex, urban–rural area, and physical activity (PA). This study included data from physical examinations of an entire population of primary school children from Split-Dalmatia County (Croatia) over a period of 10 years. There were 103,804 students from the first, fifth, and eighth grades who participated in the analysis. During the pandemic, the BMI of all the students increased, except for eighth-grade girls. Generations of eighth graders have had significantly different BMIs throughout the past decade. We found that first graders were overweight and obese in urban areas, while in rural areas, this problem was experienced by upper grades. Girls were more overweight and obese in the first and eighth grades, but boys experienced this more in the fifth grade. Reduced PA influenced an increase in BMI in both sexes, especially in girls. During the pandemic, this situation became worse. This study could be useful to experts for the creation of new policies for Split-Dalmatia County and surrounding regions that are similar economically and culturally.

## 1. Introduction

The epidemic of childhood obesity has reached a global scale in recent decades, posing a severe public health problem and a significant challenge to the scientific community [1,2,3,4]. The rising prevalence of childhood obesity in some Mediterranean countries, including Split-Dalmatia County, is a cause for concern [5,6]. Children who are overweight or obese and less physically active are more likely to remain obese once they reach adulthood and develop non-communicable chronic diseases [3,7,8,9]. Body mass index (BMI) is constantly increasing, so a higher level of prevention is needed to reduce the short- and long-term health impacts [10,11,12].

Differences in the prevalence of obesity may be caused by sex-related influences, and research claims that there is an increase by generation and age [13,14]. Research has shown that Croatia has one of the highest prevalences of overweight and obese children among European countries, and this problem is most common among boys living in the Adriatic region, where every third child is affected by this problem [15,16]. Research shows that BMI in children also depends on urbanization level [17,18]; children from rural areas have greater problems with overweight and obesity [19,20]. On 11 March 2020, the World Health Organization declared COVID-19 to be a pandemic [21]; children’s BMI increased further from this point [22,23]. Croatia adopted measures as soon as possible, like other European countries, including closing borders, limiting social interactions, and creating isolation wards within hospitals for patients with COVID-19 [24,25]. In mid-March 2020, schools in Croatia started online classes [26]. Due to the COVID-19 prevention measures, reduced physical activity (PA) and unhealthy diet proved to be key factors that negatively affected body weight [27,28,29]. One study concluded that during the COVID-19 pandemic, there was substantial weight gain across weight and age groups for all children, reflected by an increase in mean BMI-SDSs and a positive trend in weight gain patterns [30]. BMI-SDSs are measured values of BMI standardized into standard deviation scores (SDS) with respect to reference populations [31]. BMI-SDSs provide a normalized measurement for the degree of obesity in children and young people, indicating to what degree an individual’s BMI lies above or below the median BMI value [32]. Other recent studies have shown that the COVID-19 pandemic has resulted in lifestyle changes that are associated with weight gain, which can lead to obesity [33,34,35]. The onset of the pandemic imposed changes in dietary and PA patterns that led to the development of a higher BMI [36]. Access to food changed during the COVID-19 pandemic, leading to less healthy diets with higher calorie intake and lower-quality diets [29]. The pandemic worsened the trend of inactivity [37], which was alarming even before the pandemic [38]. Croatian children gained too much weight during the quarantine period due to sedentary behavior and fewer activities being carried out outside the home [16]. Therefore, the COVID-19 lockdown has led to negative lifestyle changes and an increase in BMI among children [39].

BMI is one way to measure obesity in a population and can be calculated by mathematical operations that use height and weight values to assess nutritional status and indirect assessment of health status [40,41]. Although BMI itself is not the gold standard for nutritional assessment, due to the unavailability and cost of the techniques that directly measure body fat, BMI is accepted as the clinical standard in children aged two and older [42]. Therefore, it can be used to monitor trends over a certain period. For this reason, the increasing prevalence of obesity underscores the need for continued attention to BMI monitoring and identification to address this problem [43]. For health systems to monitor the health status of children, the School and Adolescent Medicine Service of the Teaching Institute for Public Health (PHI) in Croatia conducts physical examinations following a plan and program of health protection measures for schoolchildren adopted by the Ministry of Health. The purpose of the examination is to monitor the growth, development, and nutritional status of children and to prevent and detect various diseases early [42].

The main aim of this research was (I) to examine whether the mean BMI values reported during the period of the COVID-19 pandemic (2020–2022) were significantly higher than the mean BMI values predicted by the trend in the pre-pandemic period (2012/2013–2019). The additional aims of this research were to detect (II) whether there was a difference in the mean value of BMI among generations in the last 10 years, (III) whether there were differences between urban and rural areas in BMI categories in the pre-pandemic and pandemic periods (IV), whether there were differences in relation to sex in BMI categories in the pre-pandemic and pandemic periods, and (V) whether there were differences in relation to PA in BMI before the pandemic and during the pandemic period.

## 2. Materials and Methods

### 2.1. Study Design

This was a retrospective study. The School and Adolescents Medicine Services in Croatia conduct preventive healthcare measures for children from the time they enter the first grade of primary school until the end of their schooling. During that period, children undergo physical examinations before being enrolled into the first grade of primary school and then again in the fifth grade and eighth grade. An analysis of the entire population of primary school children from Split-Dalmatia County (Croatia) who were physically examined in the period 2012–2022 was carried out. The analysis included data from physical examinations conducted over a period of 10 years. The data were collected by the School and Adolescents Medicine Service of the PHI of Split-Dalmatia County. The entire population was included in the study. Split-Dalmatia County is the largest county in the south of the Republic of Croatia and the second largest in the country in terms of population and area. 

### 2.2. Participants

A total population sampling was applied. The participants were not individually monitored through primary school; the sampling was independent. The analysis included all children within the first, fifth, and eighth grades who completed a physical examination from 2012/2013 to 2021/2022 in Split-Dalmatia County (Croatia). The grades represented 3 groups. The criteria for the inclusion of participants in the research were that the students had to be 6–15 years old, and they must have undergone a regular physical examination. When the students started school, they had to be 6 years old to be included in the research. The exclusion criteria were as follows: (I) children who were repeating a grade or delayed starting school; (II) children whose values of body weight, body height, and body mass index were not recorded; (III) children who had comorbidities; (IV) children who had a deformity, impairment, disorder, disturbance, or disability; (V) children who took drugs that could cause weight gain; and (VI) other conditions that might have made the participant a poor candidate for this research. All students who met the criteria were included. We could consider all criteria except III, V, and partially IV. The reason for this was that over the last 10 years, medical teams have not thoroughly recorded children’s comorbidities, medications, deformities, impairments, disorders, disturbances, or disabilities. We could only consider IV partly because this criterion indicated whether an individual was an immobile child or a child with a disability. The first- and fifth-grade students of 2012/2013 were not included in the analysis. The reason for this was that these first- and fifth-grade students completed their physical examination in the winter semester, and the Complete.Prevention software (Version number 3.0.0.1) was released in the summer semester. Complete data were entered only for eighth graders for 2012/2013 because they performed physical examinations in the summer semester. Considering the other generations and grades with a much greater number of participants, we had to exclude them due to the VI exclusion criteria. Accordingly, this analysis covered 9 years for the first and fifth grades and 10 years for the eighth grade. All data were provided to us by a governmental institution (the PHI of Split-Dalmatia County), which conducts physical examinations as a part of national preventive measures. The raw data had 122,939 participants. After meeting the criteria, 19,135 students were excluded, and 103,804 students were included in the analysis. Detailed inclusion and exclusion criteria can be seen in the participant flow diagram (Supplementary Figure S1).

Before starting this research, the protocol of this study was registered on the Open Science Framework. The link to access the registration is as follows: study registration. The study was conducted in accordance with the principles of the Declaration of Helsinki. The research was also conducted in accordance with Croatian laws, namely the Health Care Act (Official Gazette 100/18) and the Act on the Protection of Patients’ Rights (Official Gazette 169/04, 37/08). Consent for the research to be conducted was requested and obtained from the Ethics committee of the PHI of Split-Dalmatia County (Class: 007-05/23-01/001, No: 2181-103-11-23-002, approved on 6 April 2023). The data are owned by the Teaching Institute for the Public Health of Split-Dalmatia County; therefore, parental consent was not required. The data were obtained entirely anonymously, so the identity of the participants was not known. 

### 2.3. Data Collection and Description

Data had already been collected by the PHI of Split-Dalmatia County for the period from 2012/2013 to 2021/2022 (10 years). The total number of teams of the School and Adolescent Medicine Service of Split-Dalmatia County that collected data was 22. The teams collected the data of all children from all 96 schools in Split-Dalmatia County. The data were collected by teams of medical doctors specializing in school medicine who were additionally educated and trained every year to perform physical examinations. The data were contained in the Complete.Prevention software. The software was used by all PHIs in the Republic of Croatia so that school medicine specialists could store examination data in one place. Data were extracted from the Complete.Prevention software into an Excel spreadsheet. 

### 2.4. Entry Variables

During each measurement, body height and body weight were taken for each student. BMI was then calculated for each child. Based on Croatian reference values for BMI in children, underweight, normal weight, overweight, and obese categories were defined [44]. The entry variables used in this research were as follows: general: date of birth (date), date of measurement (date), generation/school year (2012–2022), grade (1/5/8), age (years), sex (boys/girls), and place of residence categories (urban/rural); morphologic status: body weight (kg), body height (cm), weight-for-age percentile (%), and height-for-age percentile (%); and PA (status): inactive, irregularly active, and active; in the same variable, it was written whether they had any child comorbidities, disease, deformity, impairment, disorder, disturbance, or disability and whether this was the reason for not participating in PA. Such participants were excluded from this study. 

### 2.5. Statistical Analysis

Descriptive statistics parameters were calculated. (I) Based on the value of the BMI in the period from 2013 to 2019 (2012–2019 for eighth grades), the trend function was calculated. Based on the obtained function, the theoretically expected values of the body mass index for the period 2020–2022 were determined. The differences between the defined values (empirical mean) and the values obtained by measurement in the pandemic (trend mean) were determined by applying the *t*-test. (II) The statistical significance of BMI values per generation was tested by a two-factor ANOVA according to the 9 × 2 model (10 × 2 for 8th graders) with categorical predictors of sex and generation. As a part of the analysis, the main and interaction effects of 9 × 2 (and 10 × 2) ANOVA (sex and generation and interaction effects of sex and generation) were calculated. Univariate and multivariate ANOVAs were also added. A test of the SS of the full model versus the SS of the residual was performed to assess the significance of the regression model. The statistical significance of the differences in the arithmetic means of individual subsamples under the influence of the generator of significant effects was determined using the Bonferroni test. (III) Differences between urban and rural areas were calculated using the *t*-test. (IV) Differences between sexes were calculated using the χ^2^ test. (V) Differences between BMI and PA were calculated using an ANOVA and the Bonferroni test.

Since the participants were divided into 3 groups, each aim was analyzed separately. Statistical significance was set at *p* < 0.05. The STATISTICA 14.0 program was used for data processing.

## 3. Results

This research involved 103.804 students aged 5.9 to 15.0 years old who were organized into three groups according to the measurements of the first (5.9 to 7.3 years old, 35%), fifth (10.4 to 12.0 years old, 28.9%), and eighth grades (13.4 to 15.0 years old, 36.1%). The participants were 50.2% boys and 49.8% girls. Furthermore, 66.9% of the participants lived in urban areas, while 33.1% resided in rural areas. Regarding BMI categories, 2.9% were underweight, 66.3% had a healthy weight, 17.0% were overweight, and 13.8% were obese. Most students were physically inactive (51.7%), while 44.6% were active, and only 3.7% were irregularly active. A description of the selected characteristics is shown in Table 1.

### 3.1. Comparing Pre-Pandemic (2012/2013–2019) and Pandemic Period (2020–2022) BMI

Based on the data collected in the years preceding the COVID-19 pandemic, a linear trend of BMI values was determined. By applying the thus obtained function, the approximate values of BMI in the 2020/2021 and 2021/2022 generations were determined. Table 2 presents the difference between trend projection and empirical data. The *t*-test showed that there were differences between the empirical mean (during pandemic) and the trend mean (pre-pandemic) of the BMI variable. This was significant for first-grade boys and girls during both years of the pandemic (2020/2021 and 2021/2022), fifth-grade boys and girls in 2020/2021, and eighth-grade boys in 2020/2021 and for girls during both years of the pandemic (2020/2021 and 2021/2022).

**Figure 1:**
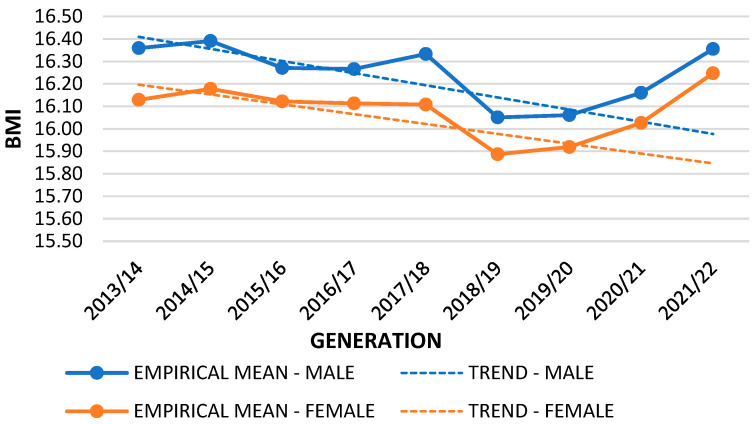
Empirical and trend mean comparison; variable: BMI; sample: 1st grade.

**Figure 2:**
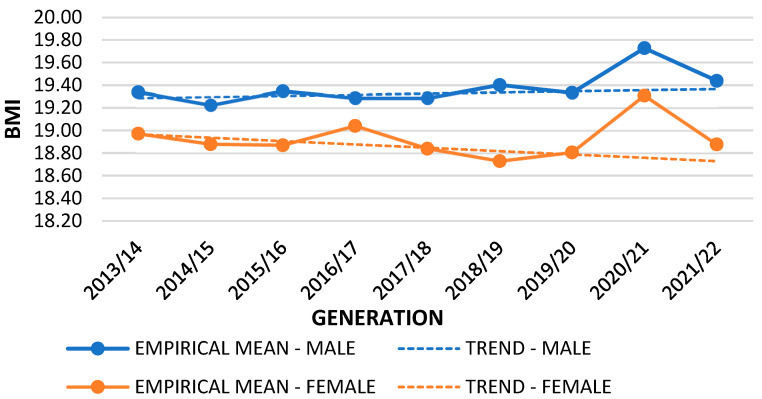
Empirical and trend mean comparison; variable: BMI; sample: 5th grade.

**Figure 3:**
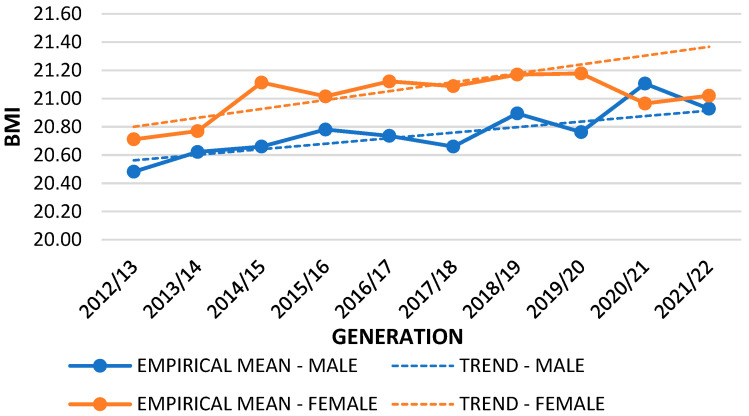
Empirical and trend mean comparison; variable: BMI; sample: 8th grade.

Differences between empirical and mean trend values are shown for the first (Figure 1), fifth (Figure 2), and eighth grades (Figure 3). The difference in the graphs between the empirical mean line (solid lines) and the trend mean line (dashed lines) represents the *t*-test difference between the lines and the measurement points. The mean trend decreased for all first graders. A slight increase is shown for boys and a slight decrease for fifth-grade girls, while for eighth graders, there was an increase in relation to both sexes. Boys had higher BMI values in the first and fifth grades, while girls had higher values in the eighth grade, except in 2020/2021, when the pandemic period began. There was a visible increase in BMI in first graders of both sexes in 2021/2022. In 2018/2019, there was a visible drop in BMI in first graders, but this then increased with the onset of the pandemic. In the fifth grade, the highest increase was recorded in both sexes in the first year of the pandemic (2020–2021). In eighth-grade boys, BMI values increased during the pandemic, but this increase was not significant. In girls, BMI values were significantly reduced in both years of the pandemic. The obtained results indicated a statistical deviation from the empirical results during the pandemic period.

### 3.2. BMI Per Generations in the Last 10 Years

The factor analysis of variance according to the 9 × 2 model for first and fifth graders and 10 × 2 for eighth graders with two categorical predictors (sex and generation) is presented in Supplementary Tables S1–S3. The confidence interval (CI) with a permissible error of the first type (type I error—false-positive) α = 5% indicates the range of variation within the population, i.e., the defined proportion of participants is within the given interval. Arithmetic means ($\bar{X}$), confidence intervals (CIs), and the number of participants (N) were calculated in the attached tables. Table 3 shows the univariate analysis of variance. The significance of the categorical predictors of sex and generation in the differentiation in the subsamples for all grades was determined (both *p* < 0.001). The interaction effect of the sex–generation factor contributed significantly to the defined model only in the eighth grade. From year to year, BMI was different and diverse. There was no significance regarding the BMI rate during 2012/2013–2022 in the first and fifth grades (*p* = 0.930; *p* = 0.288), while in the eighth grade, there was a significant difference (*p* = 0.006).

To analyze the overall quality of the model, a multivariate analysis of variance was performed, which indicates the significance of the defined two-factor model. Supplementary Table S4 shows the overall quality of the model. To determine the significance of the model, the F-test was applied, which was significant for each grade (all *p* < 0.001). 

To accurately analyze the differences between generations, a post hoc analysis was performed to determine quantitative differences in the arithmetic means of BMI. Bonferroni correction was implemented to eliminate false-positive findings by reducing the acceptable type I error level proportionally to the number of measurements. The Bonferroni test for first grades (Supplementary Table S5) showed that there were significant differences between the 2013/2014 generation and the 2018/2019 and 2019/2020 generations (both *p* < 0.001). Generations 2014/2015, 2015/2016, and 2016/2017 share the same results as generations 2018/2019 and 2019/2020 (all *p* < 0.001), while generations 2018/2019 and 2019/2020 show significant differences in relation to the 2021/2022 generation (*p* < 0.001), and the 2020/2021 generation indicates differences in relation to the 2021/2022 generation (*p* = 0.002). The Bonferroni test for the fifth grade (Supplementary Table S6) showed that there were significant differences between all generations (2013/2014–2021/2022) and 2020/2021 (*p* = 0.005, *p* < 0.001, *p* < 0.001, *p* = 0.004, *p* < 0.001, *p* < 0.001, *p* < 0.001, and *p* = 0.005). So, all fifth-grade generations showed significant differences only with the 2020/2021 generation. Furthermore, the Bonferroni test for the eighth grade (Supplementary Table S7) showed that there were significant differences between the 2012/2013 and 2014/2015–2021/2022 generations, excluding the 2017/2018 generation (*p* = 0.027, *p* = 0.019, *p* = 0.007, *p* < 0.001, *p* < 0.001, *p* < 0.001, and *p* < 0.001). The 2013/2014 generation showed differences with the 2018/2019–2021/2022 generations (*p* = 0.001, *p* = 0.19, *p* < 0.001, *p* = 0.020). Other generations did not show statistically significant results.

### 3.3. Urban–Rural and Sex Differences between BMI Categories

Differences in relation to urban–rural areas and sex in BMI categories are shown in Table 4, especially for the pre-pandemic and pandemic periods. The results of the χ^2^ test showed that there was a significant difference in BMI categories between urban and rural areas in all grades (all *p* < 0.001). During the pandemic, the first and eighth grades were significant (*p* < 0.001 and *p* = 0.002). Supplementary Table S8.1 (pre-pandemic) and Supplementary Table S8.2 (pandemic) represent contingency tables where the frequency of combinations of two categorical variables can be seen. In the pre-pandemic (2012/2013–2019) and pandemic period (2020–2022), students from urban areas were more likely to be overweight and obese in the first grades, while students from rural areas were more likely to have weight problems in the fifth and eighth grades. There was no significant difference for fifth graders during the pandemic.

As for sex-related differences, in the pre-pandemic period, all grades were significant (all *p* < 0.001), while in the pandemic period, there were significant differences in the first and fifth grades (*p* < 0.001 and *p* = 0.002). To comprehend who had significantly higher values in BMI categories, we also used the contingency tables (Supplementary Table S9.1 (pre-pandemic) and Supplementary Table S9.2 (pandemic)). In both periods, there were significantly more girls with overweight and obesity issues in the first and eighth grades, while boys had more weight problems in the fifth grade. In the pandemic period, the difference in relation to sex in the eighth grades was not significant. 

### 3.4. Differences between Physical Activity Status and BMI

Finally, differences between the three groups of PA status and BMI were obtained (Table 5). Significant differences existed in the pre-pandemic period in all grades (*p* = 0.033, *p* < 0.001, and *p* < 0.001). During the pandemic period, there were differences in the fifth and eighth grades (*p* = 0.009 and *p* = 0.014). Those who were more physically active had a lower BMI. Boys were generally more active than girls. The above is confirmed by the obtained percentages, which indicated a quantitative increase in physical inactivity. A detailed analysis can be seen in Supplementary Tables S10.1, S10.2 and S10.3.

## 4. Discussion

This retrospective study represents the first study monitoring BMI trends in Split-Dalmatia County and the first study to be conducted at the national level referring to the pre-pandemic and pandemic periods. This study included 103.804 participants, the entire population of primary school students in the county from 2012/2013 to 2022, except for those who did not meet the inclusion criteria.

Our findings showed that 30.8% of children in Split-Dalmatia County have had problems with overweight and obesity in the last decade. These results are not surprising because few children in Croatia regularly play sports through the education system [45]. Also, in the second CroCOSI study (2018/2019), the results showed that 36.9% of children were overweight and obese in the Adriatic region [46], while in the first study (2015/2016), the result was very similar and amounted to 34.9% [47]. This confirmed the phrase “Mediterranean paradox”, which refers to the high prevalence of overweight and obesity in an area traditionally characterized by healthy food [48].

### 4.1. Trends in Mean BMI Values in the Pandemic Period Compared to the Pre-Pandemic Period

Although a downward trend in the mean value was recorded for the first grades, there was a significant increase in BMI during the pandemic; it was the highest increase in the previous 8 years. However, this research provided insight into the impact of the pandemic on the rapid increase in BMI in children of that age [35,49,50]. Our results showed that as many as 92.7% of first-year students were inactive during the pandemic. During the pandemic measures, there was a drop in adherence to healthy behavior [29]. One study conducted during the pandemic, which included children from Dalmatia, showed that 49% of them had an inadequate Mediterranean diet [5]. Apart from the above, several studies have concluded that children in this age group from lower socioeconomic backgrounds may have limited access to unhealthy food options, leading to a lower BMI [51,52]. Also, first- and second-grade children in Croatia engaged in active play more on weekends than on weekdays [46]; with the arrival of the pandemic, we assume that they probably reduced the activities they would take part in at weekends. 

In the first year of the pandemic, we recorded the highest increase in BMI in the last 7 years among fifth graders. In this regard, as many as 20.4% of fifth graders were more inactive during the pandemic, which resulted in an increase in BMI. Similar results were recorded in an Italian study (mean age was 10.1 ± 2.3 years), where in the first year of the pandemic, values increased for both sexes, only to decrease again in 2021 [39]. Therefore, the increase in BMI in children aged 10–11 years was influenced by a combination of factors, including the consumption of energy-dense foods, sedentary behavior, lack of sleep, and recommended PA [53,54,55]. Research (mean age was 10.36 ± 0.49 years) has shown that children in this age category are at risk of developing an addiction to video games [56]. This is precisely the period when children play sports the most, so quarantine, a sedentary lifestyle, and a poor diet likely prevented children from taking part in these daily activities. A Croatian cross-sectional study (mean age 12.72 ± 1.17 years) found that rates of overweight and obesity increased among students who changed their habits, reduced PA, and increased their screen time during the COVID-19 quarantine. This study also showed that dietary habits varied in relation to sex and BMI [16].

For eighth graders, there was a significant unexpected drop in BMI values in the pandemic period among girls, while (2020–2021) there was the largest increase in the last 8 years among boys. For girls, the decline in BMI value was the largest for the last 7 years. The reason for this may be that classes in Croatia were sometimes organized online, and students were often isolated if someone from the school or class was infected with the SARS-CoV-2 virus [57]. Lockdown in Croatia ended in mid-May 2020 [58], and the eighth grade began to be measured at the beginning of October 2020, while examinations did not end until March 2021. It is possible that from the beginning of the pandemic until the physical examination, girls spent half of the day in front of a screen listening to classes. Since boys generally spend more time in front of a screen playing video games [59], we assume that they spent even more time in this way at the beginning of the pandemic. This was also confirmed in a Croatian study where the prevalence of excessive screen time was higher among boys than among girls aged 13–15 [60]. Therefore, we assume that girls used their free time in other ways during the pandemic period. Girls of that age in Croatia go out freely, meaning that they likely spend more of their free time walking outside and socializing with their peers than usual. Although the number of inactive students increased during the pandemic, free time could not be recorded.

However, the reasons for these trends were not entirely clear and could have been influenced by various factors. It is necessary to further investigate this phenomenon.

### 4.2. BMI Trends of Children in Split-Dalmatia County over a Period of 10 Years

The 10-year trend of BMI in children differs from year to year for all age groups. Thus, the results of this research showed that there was no difference among generations in BMI according to the effect of the sex–generation interaction in the first and fifth grades. A significant difference existed only among eighth-grade students. 

Several factors may contribute to the downward trend in BMI for first graders. One study found that BMI trends in Europe stabilized in the pre-pandemic period [6]. The data obtained in the first WHO COSI survey were a call for urgent intervention by experts dealing with obesity, so the number of active children increased slightly [47]. The first grades attend their physical examination before school, so they do not have organized school sports activities. In this period, critical age begins when excessively sedentary behavior begins [61,62]. It was also interesting that there was a greater drop in BMI in 2018/2019 for both sexes. In the summer of 2018, Croatia achieved its greatest sporting result in history, namely second place at the FIFA World Cup in Russia. The success of the Croatian team had significant benefits [63]. We assume that due to the success in 2018, there was an increase in the number of children attending sports clubs. Most children start playing sports at this age [64]. In the following months, the National Sports Program 2019–2026 was written [45], and then the most profitable Ministry of Tourism merged with the Ministry of Sports [65]. With the new National Sports Program, the Central State Sports Office suggested that the Government of the Republic of Croatia implement programs to meet public needs related to the financing of sports for children, especially the sports activities of the Croatian School Sports Association and the construction of new sports facilities [45]. 

For fifth graders, there was a slight increase in the mean trend for boys and a slight decrease for girls. Boys of that age may be replacing PA with more screen time than usual, such as playing video games, and we assume that a possible decrease in PA occurred, with this being the reason for the slightly upward BMI trend [38]. Girls obtain a perception of body image at the age of 9–12 [66], and one study (mean age 10.53 ± 0.84 years) showed that girls desire a slimmer figure than boys [67]. This could be the reason for the slight decrease in the BMI trend in girls. Inactive students had a significantly higher BMI than active students. 

For the eighth grade, the mean trend increased significantly. In the pre-pandemic period, girls were less physically active and had higher BMI values, which could be associated with a significant upward trend. One study showed similar results that boys of that age are generally more active than girls in the Mediterranean [68]. In those years, PA declines in both sexes, but it seems to decline faster in girls [69], so we assume that this is related to the earlier biological maturation of girls compared to boys in the eighth grade. Girls enter puberty earlier and gain more fat [70]. Girls at that age are less satisfied with life, have less desire to compete, and a greater fear of failure than boys; additionally, research has also shown that menstruation, social problems, lack of support, and a poorer environment in girls have a bad effect on PA [71,72]. We assume that since girls enter puberty earlier and mature sooner, they already start questioning their future adult life, while boys of that age are considered immature and still interested in children’s games.

### 4.3. Urban–Rural Differences in Relation to BMI Categories in Split-Dalmatia County in the Pre-Pandemic and Pandemic Periods

As for first-grade students, we assume that children in urban areas in Croatia play alone outside less at that age, while children in rural areas are freer and safer. Most likely, parents from urban areas have much less time to spend with their children outside. Although there is an obligation to organize sports activities as a part of preschool education, it is worrying that, in practice, this is often not carried out systematically and regularly [45]. In Croatia, children in the first grade of primary school start partaking in organized physical activities. First-grade students participated very poorly in PA in the pre-pandemic period (78.2%), and this was exacerbated in the pandemic period (92.7%). This is a possible reason why first graders from urban areas had greater problems with body weight during this timeframe.

An increase in overweight and obesity among students in the upper grades of primary school in rural areas was observed due to the lack of organized activities within educational institutions and sports clubs. One study found that children in rural areas were 26% more likely to be obese compared to children in urban areas [19]. Another study showed that the difference in nutritional status between rural and urban areas in Croatia disappeared over time and that urban children had a lower percentage of body fat [73]. The results of one study involving participants aged 12–17 further corroborated findings on low adherence to the Mediterranean diet. Despite the region’s historical dietary practices, “snacks” and “fast food” were the major contributors to energy intake, signifying a transition from traditional to Westernized dietary patterns in the Mediterranean area. This shift suggests that adherence to Mediterranean-like diets is not solely linked to geographical location and might contribute to the rising prevalence of overweight and obesity in children from Mediterranean EU countries [73]. Also, rural children have more emotional behavior in overeating [74]. This could especially come to the fore in the rural areas in the pre-pandemic period in the Dalmatian Hinterland. It could be assumed that, in the Dalmatian Hinterland, less “fast food” is eaten, but probably more fatty food is consumed. Frequent celebrations and gatherings are traditional in these regions. Therefore, fried and grilled dishes with much oil and fat are often eaten. Children acquire habits from an early age; therefore, it is assumed that they have a higher chance of becoming obese. Rural areas have fewer accessible sports facilities. This can result in reduced opportunities for regular exercise. Generally, children in rural areas have fewer opportunities to engage in some kind of organized PA due to the weak population of the area and long distances between places of residence [75]. This suggests that the environment in which children grow up may play a role in their nutritional status.

### 4.4. Sex Differences in Overweight and Obesity in Split-Dalmatia County in the Pre-Pandemic and Pandemic Periods

Our results showed that boys were more physically active in all grades, which could be related to the fact that girls in the first and eighth grades had more weight problems. Girls in the first grade were more overweight and obese because they participated in less sports than boys [46]. As for fifth graders, one Croatian study showed that the differences in PA among fifth-grade students were the smallest in relation to sex [46]. Also, in most countries with a higher average income, boys are more likely to be obese than girls [20], and Croatia belongs to that group. These results suggest that sex differences in children’s BMI categories may vary depending on the community being studied, parental influence [14], and lifestyle habits [76]. Similar results were shown by studies outside Europe [77,78]. In the eighth grade, the percentage of overweight and obese boys increased during the pandemic, while the percentage of girls decreased slightly but was still higher. One study showed that parents will limit unsupervised outdoor play much more for girls than for boys [79], and we suppose that this is also the case in Croatia. We assume that the higher percentage of girls is the result of earlier biological maturation and puberty in the pre-pandemic period, and the lower percentage in the pandemic occurred due to the perception of body image. 

### 4.5. Physical Activity Status Differences in Relation to BMI in Split-Dalmatia County in the Pre-Pandemic and Pandemic Periods

PA has decreased significantly in Split-Dalmatia County over the last decade. More than half of primary school students (51.7%) in the county were inactive in the last 10 years, which has led to an increase in BMI. The increase in inactivity reached an alarming state during the pandemic period, mostly among first graders. A recent study showed that lower PA in girls could be explained by less PA at school and participation in community sports [38]. In European Mediterranean countries, the PA of children has decreased in the last 20 years [80], but this slightly improved for Croatia once the country joined the WHO COSI study [81]. During the pandemic, the PA of children in Croatia decreased by 30%, which resulted in an increased risk of obesity [82]. Due to the promotion of PA in 2021, the Croatian PHI, in cooperation with the county PHIs, implemented the activities “Walking for Health” and “Volunteering in Parks” [83]. This is probably why the BMI of the upper grades decreased slightly in 2022. In this regard, the lack of PA influenced the increase in BMI in Split-Dalmatia County as well.

### 4.6. Strengths and Limitations of the Study

This study has dealt with a topic that is contemporary and relevant. The main strength of this study refers to the sampling of the total population, which has strengthened the generalizability of the results. This approach allowed us to gather data from every individual in the population, ensuring comprehensive insights into the state of the entire region. The detailed study design enabled a thorough data analysis to be carried out. To prevent omissions, this included the precise elaboration of each research phase, data collection methodology, inclusion and exclusion criteria, and statistical analysis approaches. Also, as a part of the research, the first degree of sensitivity of the applied measures was determined, and this had a positive effect on the internal validity of the study. The study used appropriate statistical methods to analyze the data and draw significant conclusions. This included the use of statistical tests to assess the quality of the model, control for potential confounding factors, and better assess the significance of the findings. Also, this article has been written following the guidelines of the STROBE checklist for observational studies. The research is applicable to other Mediterranean regions that share the same economic and cultural aspects. Therefore, it can refer to a large part of Croatian schoolchildren because all counties in the country share the abovementioned aspects.

This study had several limitations. This study was of a retrospective nature, meaning that the researchers relied on existing data and could not control the data collection process. This may have led to biases and limitations being introduced to the data. This study did not consider other variables that may influence body weight, sedentary lifestyle, dietary habits, or socioeconomic variables. Specific socioeconomic, environmental, or cultural aspects that were particular to the study area may have had an impact on the results. Furthermore, the PHI of Split-Dalmatia County had data in three points, so there was a theoretical possibility that some trends occurred in periods not covered by the three measurement points. Some participants performed the physical examination in the morning, whereas it was conducted for some in the afternoon. Some students may have been born earlier in the year, and some may have been born near the end of the same year but attended the same grade of primary school and were the same age at the time of the physical examination. Also, not all teams of the School and Adolescent Medicine Service started their physical examinations at the same time. In that sensitive period, a few months can mean a lot. We lacked data on the PA status of some participants, so we could only perform analyses based on what we had. Considering the large number of participants, we expect that potential deviations from the initial value will be neutralized.

### 4.7. Suggestions for Curbing the Prevalence of Overweight and Obesity

Understanding how the pandemic has affected children’s health and wellbeing is critical to guiding public health initiatives and policies. The findings of this study suggest that BMI trends in children can vary depending on various factors. It is crucial to monitor the BMI trends of children and take appropriate measures to prevent overweight and obesity, which can lead to various health problems. To prevent the prevalence of overweight and obesity among children, better cooperation between the sports and health sectors is needed. Perhaps extracurricular sports activities should be implicated in school performance to further encourage children to engage in PA. Political society should finance sports organizations even more, especially school sports, to help promote a healthy lifestyle. It is necessary to encourage and create conditions for more children to participate in a system of exercises in educational institutions. We place a special emphasis on Scout movements that organize trips within nature and influence socialization, education, environmental protection, children’s development, and the creation of healthy lifestyle habits. The findings of this study will serve as a basis for future research on the influence of the COVID-19 pandemic on children’s health.

## 5. Conclusions

In conclusion, we provide an overview of BMI trends in Split-Dalmatia County in the last 10 years among primary school children. The analysis of differences in trends showed that the pandemic influenced the increase in BMI. The additional value of this study is that BMI was investigated for the first time in Split-Dalmatia County. Addressing childhood obesity requires a multisectoral approach through health and education policies. We believe that policies promoting a healthy lifestyle for children throughout their education should be implemented much more to raise the awareness of children and parents about the importance of engaging in PA and consuming healthy food. Therefore, the focus should be on the implementation of new policies to prevent the prevalence of obesity.

## Figures and Tables

**Table 1:** Descriptive statistics of selected characteristics.

Variable	
BMI	(means ± SD)
BMI total	18.730 ± 3.711
BMI pre-pandemic	18.716 ± 3.678
BMI during pandemic	18.788 ± 3.843
Sex	*n* (%)
Boys	52,123 (50.2)
Girls	51,681 (49.8)
Age group (years)	*n* (%)
1st grade (5.9–7.3)	36,361 (35.0)
5th grade (10.4–12.0)	29,950 (28.9)
8th grade (13.4–15.0)	37,493 (36.1)
Area of residence *	*n* (%)
Urban	69,437 (66.9)
Rural	34,367 (33.1)
BMI categories	*n* (%)
Underweight	3006 (2.9)
Healthy weight	68,870 (66.3)
Overweight	17,610 (17.0)
Obesity	14,318 (13.8)
Physical activity status	*n* (%)
Inactive	40,297 (51.7)
Irregularly active	2917 (3.7)
Active	34,793 (44.6)

Standard deviation (SD). * Area of residence according to “The Model of Differentiation of Urban, Rural and Semi-Urban Settlements in the Republic of Croatia”, which can be found on the website of the Croatian Bureau of Statistics via the link: “https://podaci.dzs.hr/media/st2d33m1/metod_67.pdf (accessed on 25 June 2023)”.

**Table 2:** *T*-test between empirical mean (during pandemic) and trend mean (pre-pandemic); variable: BMI.

	Sex	Generation	Empirical Mean	Trend Mean	N	*df*	*t*-Test	*p*
1st	Boys	2020/2021	16.16	16.03	1888	3774	1.66	0.048
		2021/2022	16.36	15.98	1949	3896	4.98	<0.001
	Girls	2020/2021	16.03	15.89	1839	3676	1.79	0.037
		2021/2022	16.25	15.85	1809	3616	5.02	<0.001
5th	Boys	2020/2021	19.73	19.36	1057	2112	2.29	0.011
		2021/2022	19.44	19.37	1582	3162	0.00	0.050
	Girls	2020/2021	19.31	18.76	973	1944	3.29	<0.001
		2021/2022	18.88	18.73	1613	3224	1.24	0.108
8th	Boys	2020/2021	21.11	20.88	1902	3802	1.91	0.028
		2021/2022	20.93	20.92	1797	3592	0.10	0.460
	Girls	2020/2021	20.97	21.30	1881	3760	−3.02	<0.001
		2021/2022	21.02	21.37	1953	3904	−2.98	<0.001

Degrees of freedom (*df*) and statistical significance (*p*).

**Table 3:** Univariate results; dependent variable: BMI; sigma-restricted parameterization, effective hypothesis decomposition; 1st-, 5th-, and 8th-grade students.

	Effect	*df*	F	*p*
1st	Intercept	1	1,925,332.90	<0.001
	Sex	1	52.471	<0.001
	Generation	8	11.831	<0.001
	Sex × Generation	8	0.383	0.930
5th	Intercept	1	889,687.23	<0.001
	Sex	1	124.121	<0.001
	Generation	8	3.977	<0.001
	Sex × Generation	8	1.210	0.288
8th	Intercept	1	1,291,463.00	<0.001
	Sex	1	47.008	<0.001
	Generation	9	5.764	<0.001
	Sex × Generation	9	2.580	0.006

Degrees of freedom (*df*), F-statistics (F), and statistical significance (*p*).

**Table 4:** Chi-square test 2 × 3 urban–rural areas between BMI categories and chi-square test 2 × 3 sexes between BMI categories; 1st, 5th, and 8th grade in the pre-pandemic and pandemic periods.

		Pre-Pandemic			During Pandemic		
		χ^2^	*df*	*p*	χ^2^	*df*	*p*
1st	Urban–rural	45.649	3	<0.001	38.093	3	<0.001
5th		24.496	3	<0.001	6.290	3	0.098
8th		19.038	3	<0.001	14.686	3	0.002
1st	Sex	67.339	3	<0.001	18.214	3	<0.001
5th		58.231	3	<0.001	14.557	3	0.002
8th		89.186	3	<0.001	1.960	3	0.581

BMI categories: underweight, healthy weight, overweight, and obesity.

**Table 5:** Analysis of variance; dependent variable: BMI; grouping variable: PA status; 1st, 5th, and 8th grade in the pre-pandemic and pandemic periods.

	Pre-Pandemic			During Pandemic		
	*df*	F	*p*	*df*	F	*p*
1st	2	3.409	0.033	2	1.056	0.348
5th	2	19.057	<0.001	2	4.683	0.009
8th	2	28.851	<0.001	2	4.268	0.014

Degrees of freedom (*df*), F-statistics (F), and statistical significance (*p*).

## Data Availability

The obtained raw data are available under the registration of the study on the Open Science Framework under files. The registration link: https://osf.io/r3v9u/files/osfstorage. Further inquiries may be directed to the corresponding author.

## Supplementary Materials

The following supporting information can be downloaded at https://www.mdpi.com/article/10.3390/nu16010050/s1, Figure S1: Participant flow diagram. Table S1: Effect: SEX × GENERATION; unweighted means, current effect, effective hypothesis decomposition; 1st-grade students. Table S2: Effect: SEX × GENERATION; unweighted means, current effect, effective hypothesis decomposition; 5th-grade students. Table S3: Effect: SEX × GENERATION; unweighted means, current effect, effective hypothesis decomposition; 8th-grade students. Table S4: Test of SS whole model vs. SS residual; dependent variable: BMI; 1st-, 5th-, and 8th-grade students. Table S5: Bonferroni test; probabilities for post hoc tests error: between MS = 4.924, *df* = 36352; dependent variable: BMI; effect: GENERATION; 1st-grade students. Table S6: Bonferroni test; probabilities for post hoc tests error: between MS = 11.993, *df* = 29941; dependent variable: BMI; effect: GENERATION; 5th-grade students. Table S7: Bonferroni test; probabilities for post hoc tests error: between MS = 12.571, *df* = 37483; dependent variable: BMI; effect: GENERATION; 8th-grade students. Table S8.1: Contingency table in the pre-pandemic period for all grades; urban–rural areas in relation to BMI categories. Table S8.2: Contingency table in the pandemic period for all grades; urban–rural areas in relation to BMI categories. Table S9.1: Contingency table in the pre-pandemic period for all grades; sexes in relation to BMI categories. Table S9.2: Contingency table in the pandemic period for all grades; sexes in relation to BMI categories. Table S10.1: Descriptive statistics of the PA status; 1st, 5th, and 8th grade in the pre-pandemic and pandemic periods. Table S10.2. Post hoc tests; Bonferroni tests; dependent variable: BMI; effect: PA status; 1st, 5th, and 8th grade in the pre-pandemic and pandemic periods. Table S10.3. Descriptive statistics of the PA status in relation to sex; 1st, 5th, and 8th grade.
