# Supplementary material for: Body Mass Index Trends before and during the COVID-19 Pandemic in Primary School Students in Split-Dalmatia County, Croatia: A Retrospective Study
Source: Nutrients. 2023 Dec 22;16(1):50. doi: 10.3390/nu16010050 (PMC10780733; doi:10.3390/nu16010050)
Supplement: Supplementary file 1 [file nutrients-16-00050-s001.zip › nutrients-2754801-supplementary.pdf]

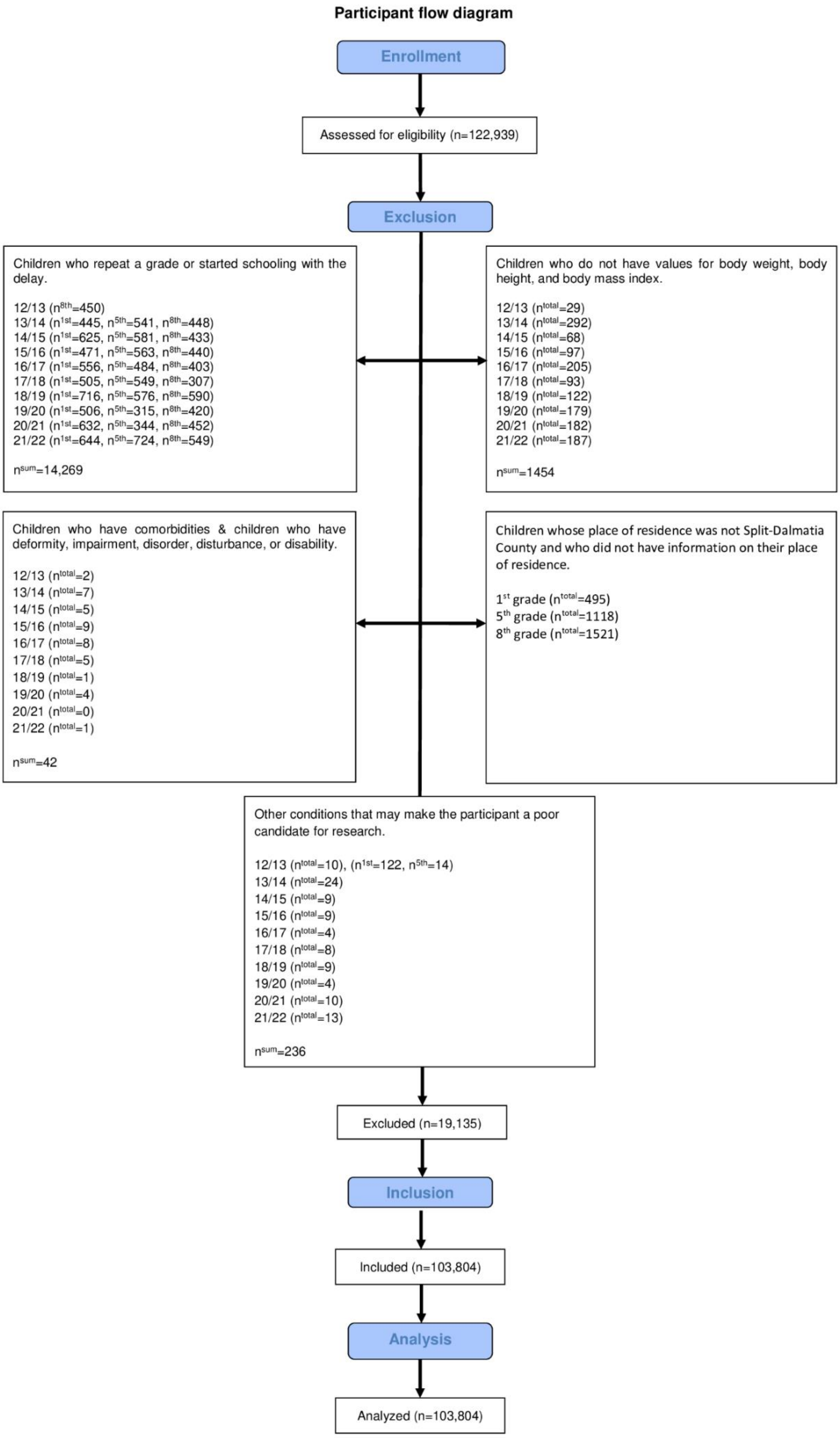

Figure S1: Participant flow diagram

Supplemental Table S1. Effect: SEX x GENERATION; unweighted Means, current effect, effective hypothesis decomposition; BMI in 1st graders

SEX	Generation	Mean	Std. Err.	-95.00%	+95.00%	N
**Boys**	2013/14	16.13	0.05	16.03	16.23	1925
	2014/15	16.18	0.05	16.08	16.27	2134
	2015/16	16.12	0.05	16.03	16.22	2128
	2016/17	16.11	0.05	16.02	16.21	2057
	2017/18	16.11	0.05	16.01	16.20	2027
	2018/19	15.89	0.05	15.79	15.99	1882
	2019/20	15.92	0.05	15.82	16.02	1933
	2020/21	16.03	0.05	15.92	16.13	1839
	2021/22	16.25	0.05	16.14	16.35	1809
**Girls**	2013/14	16.36	0.05	16.26	16.46	2029
	2014/15	16.39	0.05	16.30	16.49	2112
	2015/16	16.27	0.05	16.18	16.36	2137
	2016/17	16.27	0.05	16.17	16.36	2262
	2017/18	16.33	0.05	16.24	16.43	2174
	2018/19	16.05	0.05	15.95	16.15	2048
	2019/20	16.06	0.05	15.96	16.16	2028
	2020/21	16.16	0.05	16.06	16.26	1888
	2021/22	16.35	0.05	16.26	16.45	1949
	F (8.36343) = 0.383			p = 0.930		

Supplemental Table S2. Effect: SEX x GENERATION; unweighted Means, current effect, effective hypothesis decomposition; BMI in 5th graders

Sex	Generation	Mean	Std. Err.	-95.00%	+95.00%	N
**Boys**	2013/14	18.97	0.08	18.81	19.13	1725
	2014/15	18.88	0.09	18.71	19.05	1645
	2015/16	18.87	0.08	18.71	19.03	1788
	2016/17	19.04	0.08	18.89	19.19	1942
	2017/18	18.84	0.08	18.68	19.00	1778
	2018/19	18.73	0.08	18.57	18.88	1869
	2019/20	18.80	0.08	18.64	18.97	1691
	2020/21	19.31	0.11	19.09	19.52	973
	2021/22	18.88	0.09	18.71	19.04	1613
**Girls**	2013/14	19.34	0.08	19.18	19.50	1830
	2014/15	19.22	0.09	19.05	19.40	1561
	2015/16	19.35	0.08	19.19	19.50	1859
	2016/17	19.29	0.08	19.13	19.44	1866
	2017/18	19.29	0.08	19.13	19.44	1829
	2018/19	19.40	0.08	19.24	19.56	1740
	2019/20	19.33	0.09	19.16	19.50	1602
	2020/21	19.73	0.11	19.52	19.94	1057
	2021/22	19.44	0.09	19.27	19.61	1582
	F (8.29932) = 1.210			p = 0.288		

Supplemental Table S3. Effect: SEX x GENERATION; unweighted Means, current effect, effective hypothesis decomposition; BMI in 8th graders

SEX	Generation	Mean	Std. Err.	-95.00%	+95.00%	N
**Boys**	2012/13	20.48	0.09	20.31	20.66	1540
	2013/14	20.62	0.08	20.47	20.77	2086
	2014/15	20.66	0.08	20.51	20.81	2082
	2015/16	20.78	0.08	20.62	20.94	1913
	2016/17	20.74	0.08	20.58	20.89	1954
	2017/18	20.66	0.09	20.48	20.84	1469
	2018/19	20.90	0.08	20.74	21.05	1957
	2019/20	20.76	0.08	20.60	20.92	1870
	2020/21	21.11	0.08	20.95	21.27	1902
	2021/22	20.93	0.08	20.76	21.09	1797
**Girls**	2012/13	20.71	0.08	20.55	20.88	1756
	2013/14	20.77	0.08	20.61	20.93	1995
	2014/15	21.11	0.08	20.96	21.27	2099
	2015/16	21.02	0.08	20.86	21.17	1976
	2016/17	21.12	0.08	20.96	21.28	1845
	2017/18	21.09	0.09	20.91	21.26	1566
	2018/19	21.17	0.08	21.01	21.33	1875
	2019/20	21.18	0.08	21.02	21.33	1977
	2020/21	20.96	0.08	20.80	21.13	1881
	2021/22	21.02	0.08	20.86	21.18	1953
	F (9.37473) = 2.580				p = 0.006	

Supplemental Table S4. Test of SS whole model vs. SS residual; dependent variable: BMI; 1st, 5th and 8th grade students

	Multiple R	Multiple R^2	Adjusted R^2	SS Model	Df Model	MS Model	SS Residual	Df Residual	MS Residual	F	p
**1st**	0.06	0.00	0.00	740.26	17	43.54	178703.04	36343	4.917124	8.86	< 0.001
**5th**	0.08	0.01	0.01	2047.71	17	120.45	357448.03	29932	11.94	10.09	< 0.001
**8th**	0.06	0.00	0.00	1520.33	19	80.02	470305.89	37473.00	12.55	6.38	< 0.001

Supplemental Table S5. Bonferroni test; probabilities for post hoc tests error: between MS = 4.924, df = 36352; dependent variable: BMI; effect: GENERATION; 1st grade students

		Mean Difference	95% CI for Mean Difference		SE	t	p_{bonf}
			Lower	Upper			
2013/14	2014/15	-0.037	-0.189	0.116	0.049	-0.746	1.000
	2015/16	0.051	-0.101	0.203	0.049	1.042	1.000
	2016/17	0.054	-0.097	0.206	0.049	1.111	1.000
	2017/18	0.023	-0.130	0.175	0.049	0.467	1.000
	2018/19	0.275	0.120	0.430	0.050	5.507	< 0.001
	2019/20	0.255	0.100	0.410	0.050	5.116	< 0.001
	2020/21	0.153	-0.004	0.311	0.051	3.030	0.088
	2021/22	-0.056	-0.212	0.101	0.051	-1.098	1.000
2014/15	2015/16	0.088	-0.062	0.237	0.048	1.822	1.000
	2016/17	0.091	-0.058	0.240	0.048	1.895	1.000
	2017/18	0.060	-0.090	0.209	0.048	1.233	1.000
	2018/19	0.312	0.159	0.464	0.049	6.349	< 0.001
	2019/20	0.292	0.140	0.444	0.049	5.953	< 0.001
	2020/21	0.190	0.036	0.345	0.050	3.816	0.005
	2021/22	-0.019	-0.173	0.135	0.050	-0.381	1.000
2015/16	2016/17	0.003	-0.145	0.152	0.048	0.068	1.000
	2017/18	-0.028	-0.178	0.122	0.048	-0.582	1.000
	2018/19	0.224	0.072	0.376	0.049	4.570	< 0.001
	2019/20	0.204	0.052	0.356	0.049	4.170	0.001
	2020/21	0.102	-0.052	0.257	0.050	2.059	1.000
	2021/22	-0.107	-0.261	0.047	0.050	-2.146	1.000
2016/17	2017/18	-0.031	-0.180	0.118	0.048	-0.651	1.000
	2018/19	0.221	0.069	0.373	0.049	4.517	< 0.001
	2019/20	0.201	0.050	0.352	0.049	4.116	0.001
	2020/21	0.099	-0.055	0.253	0.050	2.000	1.000
	2021/22	-0.110	-0.263	0.044	0.049	-2.218	0.956
2017/18	2018/19	0.252	0.100	0.405	0.049	5.123	< 0.001
	2019/20	0.232	0.080	0.385	0.049	4.726	< 0.001
	2020/21	0.131	-0.024	0.285	0.050	2.614	0.322
	2021/22	-0.078	-0.233	0.076	0.050	-1.575	1.000
2018/19	2019/20	-0.020	-0.175	0.135	0.050	-0.401	1.000
	2020/21	-0.122	-0.279	0.036	0.051	-2.400	0.590
	2021/22	-0.331	-0.488	-0.174	0.051	-6.533	< 0.001
2019/20	2020/21	-0.102	-0.259	0.055	0.051	-2.009	1.000
	2021/22	-0.311	-0.467	-0.154	0.051	-6.149	< 0.001
2020/21	2021/22	-0.209	-0.368	-0.050	0.051	-4.074	0.002

Standard Error (SE), t-statistics (t) and Bonferroni statistical significance (p_{bonf})

Note. Bonferroni correction was used in this analysis to account for multiple comparisons. This analysis was chosen because there were many groups with different sample sizes and because it adjusts the level of significance based on the number of tests performed.

Supplemental Table S6. Bonferroni test; probabilities for post hoc tests error: between MS = 11.993, df = 29941; dependent variable: BMI; effect: GENERATION; 5th grade students

			95% CI for Mean Difference				
		Mean Difference	Lower	Upper	SE	t	p_{bonf}
2013/14	2014/15	0.114	-0.148	0.376	0.084	1.352	1.000
	2015/16	0.048	-0.206	0.301	0.082	0.583	1.000
	2016/17	-1.382×10^{-4}	-0.251	0.250	0.081	-0.002	1.000
	2017/18	0.095	-0.159	0.349	0.082	1.163	1.000
	2018/19	0.108	-0.146	0.361	0.082	1.315	1.000
	2019/20	0.099	-0.161	0.359	0.084	1.178	1.000
	2020/21	-0.367	-0.665	-0.068	0.096	-3.804	0.005
	2021/22	0.006	-0.256	0.268	0.084	0.072	1.000
2014/15	2015/16	-0.066	-0.327	0.194	0.084	-0.793	1.000
	2016/17	-0.114	-0.372	0.143	0.083	-1.375	1.000
	2017/18	-0.019	-0.280	0.242	0.084	-0.224	1.000
	2018/19	-0.006	-0.267	0.254	0.084	-0.076	1.000
	2019/20	-0.015	-0.282	0.251	0.086	-0.178	1.000
	2020/21	-0.481	-0.785	-0.176	0.098	-4.892	< 0.001
	2021/22	-0.108	-0.376	0.161	0.087	-1.247	1.000
2015/16	2016/17	-0.048	-0.297	0.201	0.080	-0.595	1.000
	2017/18	0.048	-0.205	0.300	0.081	0.586	1.000
	2018/19	0.060	-0.192	0.312	0.081	0.738	1.000
	2019/20	0.051	-0.207	0.309	0.083	0.614	1.000
	2020/21	-0.414	-0.712	-0.117	0.096	-4.318	< 0.001
	2021/22	-0.041	-0.302	0.219	0.084	-0.494	1.000
2016/17	2017/18	0.095	-0.154	0.345	0.080	1.185	1.000
	2018/19	0.108	-0.142	0.357	0.080	1.339	1.000
	2019/20	0.099	-0.157	0.354	0.082	1.199	1.000
	2020/21	-0.366	-0.662	-0.071	0.095	-3.850	0.004
	2021/22	0.006	-0.251	0.264	0.083	0.075	1.000
2017/18	2018/19	0.012	-0.241	0.265	0.082	0.152	1.000
	2019/20	0.003	-0.255	0.262	0.083	0.042	1.000
	2020/21	-0.462	-0.760	-0.164	0.096	-4.805	< 0.001
	2021/22	-0.089	-0.350	0.172	0.084	-1.059	1.000
2018/19	2019/20	-0.009	-0.268	0.250	0.083	-0.107	1.000
	2020/21	-0.474	-0.772	-0.176	0.096	-4.935	< 0.001
	2021/22	-0.102	-0.362	0.159	0.084	-1.207	1.000
2019/20	2020/21	-0.465	-0.768	-0.162	0.098	-4.761	< 0.001
	2021/22	-0.093	-0.359	0.174	0.086	-1.077	1.000
2020/21	2021/22	0.373	0.068	0.678	0.098	3.791	0.005

Standard Error (SE), t-statistics (t) and Bonferroni statistical significance (p_{bonf})

Note. Bonferroni correction was used in this analysis to account for multiple comparisons. This analysis was chosen because there were many groups with different sample sizes and because it adjusts the level of significance based on the number of tests performed.

		Mean Difference	95% CI for Mean Difference		SE	t	p_{bonf}
			Lower	Upper			

Supplemental Table S7. Bonferroni test; probabilities for post hoc tests error: between MS = 12.571, df = 37483; dependent variable: BMI; effect: GENERATION; 8th grade students

		Mean Difference	95% CI for Mean Difference		SE	t	p_{bonf}
			Lower	Upper			
2012/13	2013/14	-0.090	-0.353	0.172	0.083	-1.087	1.000
	2014/15	-0.284	-0.545	-0.022	0.083	-3.435	0.027
	2015/16	-0.296	-0.562	-0.030	0.084	-3.526	0.019
	2016/17	-0.319	-0.586	-0.052	0.084	-3.777	0.007
	2017/18	-0.276	-0.558	0.006	0.089	-3.098	0.088
	2018/19	-0.425	-0.692	-0.159	0.084	-5.048	< .001
	2019/20	-0.372	-0.638	-0.106	0.084	-4.418	< .001
	2020/21	-0.432	-0.699	-0.164	0.084	-5.111	< .001
	2021/22	-0.372	-0.640	-0.104	0.085	-4.392	< .001
2013/14	2014/15	-0.193	-0.440	0.053	0.078	-2.479	0.593
	2015/16	-0.206	-0.457	0.046	0.079	-2.590	0.432
	2016/17	-0.229	-0.481	0.024	0.080	-2.859	0.192
	2017/18	-0.186	-0.455	0.083	0.085	-2.189	1.000
	2018/19	-0.335	-0.587	-0.083	0.080	-4.199	0.001
	2019/20	-0.281	-0.534	-0.029	0.080	-3.533	0.019
	2020/21	-0.342	-0.595	-0.088	0.080	-4.268	< .001
	2021/22	-0.282	-0.535	-0.028	0.080	-3.510	0.020
2014/15	2015/16	-0.012	-0.262	0.238	0.079	-0.156	1.000
	2016/17	-0.035	-0.286	0.216	0.079	-0.441	1.000
	2017/18	0.007	-0.260	0.275	0.085	0.087	1.000
	2018/19	-0.141	-0.392	0.109	0.079	-1.784	1.000
	2019/20	-0.088	-0.339	0.163	0.079	-1.112	1.000
	2020/21	-0.148	-0.400	0.104	0.080	-1.861	1.000
	2021/22	-0.088	-0.340	0.164	0.080	-1.105	1.000
2015/16	2016/17	-0.023	-0.279	0.233	0.081	-0.281	1.000
	2017/18	0.020	-0.252	0.291	0.086	0.229	1.000
	2018/19	-0.129	-0.384	0.126	0.081	-1.600	1.000
	2019/20	-0.076	-0.331	0.179	0.081	-0.939	1.000
	2020/21	-0.136	-0.392	0.120	0.081	-1.677	1.000
	2021/22	-0.076	-0.332	0.181	0.081	-0.934	1.000
2016/17	2017/18	0.042	-0.231	0.316	0.086	0.492	1.000
	2018/19	-0.106	-0.363	0.150	0.081	-1.311	1.000
	2019/20	-0.053	-0.310	0.204	0.081	-0.653	1.000
	2020/21	-0.113	-0.371	0.145	0.081	-1.388	1.000
	2021/22	-0.053	-0.311	0.205	0.082	-0.650	1.000

		Mean Difference	95% CI for Mean Difference		SE	t	p_{bonf}
			Lower	Upper			
2017/18	2018/19	-0.149	-0.421	0.124	0.086	-1.728	1.000
	2019/20	-0.095	-0.368	0.177	0.086	-1.109	1.000
	2020/21	-0.155	-0.429	0.118	0.086	-1.799	1.000
	2021/22	-0.095	-0.369	0.178	0.087	-1.103	1.000
2018/19	2019/20	0.053	-0.203	0.309	0.081	0.660	1.000
	2020/21	-0.007	-0.264	0.250	0.081	-0.081	1.000
	2021/22	0.053	-0.204	0.311	0.081	0.656	1.000
2019/20	2020/21	-0.060	-0.317	0.197	0.081	-0.739	1.000
	2021/22	-1.343×10^{-5}	-0.257	0.257	0.081	-1.650×10^{-4}	1.000
2020/21	2021/22	0.060	-0.198	0.318	0.082	0.734	1.000

Standard Error (SE), t-statistics (t) and Bonferroni statistical significance (p_{bonf})

Note. Bonferroni correction was used in this analysis to account for multiple comparisons. This analysis was chosen because there were many groups with different sample sizes and because it adjusts the level of significance based on the number of tests performed.

Supplemental Table S8.1. Contingency table in the pre-pandemic period for all grades; urban-rural areas in relation to BMI categories

Grade	Area		BMI Categories				Total
			Healthy weight	Overweight	Obesity	Underweight	
1	Rural	N	5726	969	2507	303	9505
		% row	60.2 %	10.2 %	26.4 %	3.2 %	100 %
	Urban	N	11600	1852	5518	401	19371
		% row	59.9 %	9.6 %	28.5 %	2.1 %	100 %
	Total	N	17326	2821	8025	704	28876
		% row	60.0 %	9.8 %	27.8 %	2.4 %	100 %
5	Rural	N	5481	1439	1061	195	8176
		% row	67.0 %	17.6 %	13.0 %	2.4 %	100%
	Urban	N	11528	2774	1836	411	16549
		% row	69.6 %	16.8 %	11.1 %	2.5 %	100 %
	Total	N	17009	4213	2897	606	24725
		% row	68.8 %	17.1 %	11.7 %	2.4 %	100 %
8	Rural	N	6952	1500	1212	346	10010
		% row	69.5 %	15.0 %	12.1 %	3.4 %	100 %
	Urban	N	14213	2948	2093	696	19950
		% row	71.2 %	14.8 %	10.5 %	3.5 %	100 %
	Total	N	21165	4448	3305	1042	29960
		% row	70.6 %	14.9 %	11.0 %	3.5 %	100 %
Total	Rural	N	18159	3908	4780	844	27691
		% row	65.6 %	14.1 %	17.3 %	3.0 %	100 %
	Urban	N	37341	7574	9447	1508	55870
		% row	66.8 %	13.6 %	16.9 %	2.7 %	100 %
	Total	N	55500	11482	14227	2352	83561
		% row	66.4 %	13.8 %	17.0 %	2.8 %	100 %

Supplemental Table S8.2. Contingency table in the pandemic period for all grades; urban-rural areas in relation to BMI categories

Grade	Area		BMI Categories				Total
			Healthy weight	Overweight	Obesity	Underweight	
1	Rural	N	1577	262	584	143	2566
		% row	61.5 %	10.2 %	22.8 %	5.6 %	100 %
	Urban	N	3058	546	1179	136	4919
		% row	62.2 %	11.1 %	24.0 %	2.7 %	100 %
	Total	N	4635	808	1763	279	7485
		% row	62.0 %	10.8 %	23.6 %	3.7 %	100 %
5	Rural	N	1079	288	254	37	1658
		% row	65.1 %	17.4 %	15.3 %	2.2 %	100 %
	Urban	N	2403	626	456	82	3567
		% row	67.4 %	17.6 %	12.8 %	2.3 %	100 %
	Total	N	3482	914	710	119	5225
		% row	66.6 %	17.5 %	13.6 %	2.3 %	100 %
8	Rural	N	1652	370	344	86	2452
		% row	67.4 %	15.1 %	14.0 %	3.5 %	100 %
	Urban	N	3601	744	566	170	5081
		% row	70.9 %	14.6 %	11.1 %	3.3 %	100 %
	Total	N	5253	1114	910	256	7533
		% row	69.7 %	14.8 %	12.1 %	3.4 %	100 %
Total	Rural	N	4308	920	1182	266	6676
		% row	64.5 %	13.8 %	17.7 %	4.0 %	100 %
	Urban	N	9062	1916	2201	388	13567
		% row	66.8 %	14.1 %	16.2 %	2.8 %	100 %
	Total	N	13370	2836	3383	654	20243
		% row	66.1 %	14.0 %	16.7 %	3.2 %	100 %

Supplemental Table S9.1. Contingency table in the pre-pandemic period for all grades; sexes in relation to BMI categories

Grade	Area		BMI Categories				Total
			Healthy weight	Overweight	Obesity	Underweight	
1	Boys	N	8885	1400	4040	465	14790
		% row	60.1 %	9.6 %	27.3 %	3.1 %	100 %
	Girls	N	8441	1421	3985	239	14086
		% row	59.9 %	10.1 %	28.3 %	1.7 %	100 %
	Total	N	17326	2821	8025	704	28876
		% row	60.0 %	9.8 %	27.8 %	2.4 %	100 %
5	Boys	N	8291	2146	1597	253	12287
		% row	67.5 %	17.5 %	12.9 %	2.1 %	100 %
	Girls	N	8718	2067	1300	353	12438
		% row	70.1 %	16.6 %	10.5 %	2.8 %	100 %
	Total	N	17009	4213	2897	606	24725
		% row	68.8 %	17.0 %	11.7 %	2.5 %	100 %
8	Boys	N	10864	2010	1484	513	14871
		% row	73.1 %	13.5 %	10.0 %	3.4 %	100 %
	Girls	N	10301	2438	1821	529	15089
		% row	68.3 %	16.2 %	12.1 %	3.5 %	100 %
	Total	N	21165	4448	3305	1042	29960
		% row	70.6 %	14.8 %	11.0 %	3.5 %	100 %
Total	Boys	N	28040	5556	7121	1231	41948
		% row	66.9 %	13.2 %	17.0 %	2.9 %	100 %
	Girls	N	27460	5926	7106	1121	41613
		% row	66.0 %	14.2 %	17.1 %	2.7 %	100 %
	Total	N	55500	11482	14227	2352	83561
		% row	66.4 %	13.8 %	17.0 %	2.8 %	100 %

Supplemental Table S9.2. Contingency table in the pandemic period for all grades; sexes in relation to BMI categories

Grade	Area		BMI Categories				Total
			Healthy weight	Overweight	Obesity	Underweight	
1	Boys	N	2408	387	871	171	3837
		% row	62.8 %	10.1 %	22.7 %	4.4 %	100 %
	Girls	N	2227	421	892	108	3648
		% row	61.1 %	11.5 %	24.5 %	2.9 %	100 %
	Total	N	4635	808	1763	279	7485
		% row	61.9 %	10.8 %	23.6 %	3.7 %	100 %
5	Boys	N	1706	479	399	55	2639
		% row	64.6 %	18.2 %	15.1 %	2.1 %	100 %
	Girls	N	1776	435	311	64	2586
		% row	68.7 %	16.8 %	12.0 %	2.5 %	100 %
	Total	N	3482	914	710	119	5225
		% row	66.6 %	17.5 %	13.6 %	2.3 %	100 %
8	Boys	N	2596	533	452	118	3699
		% row	70.2 %	14.4 %	12.2 %	3.2 %	100 %
	Girls	N	2657	581	458	138	3834
		% row	69.3 %	15.2 %	11.9 %	3.6 %	100 %
	Total	N	5253	1114	910	256	7533
		% row	69.7 %	14.8 %	12.1 %	3.4 %	100 %
Total	Boys	N	6710	1399	1722	344	10175
		% row	65.9 %	13.8 %	16.9 %	3.4 %	100 %
	Girls	N	6660	1437	1661	310	10068
		% row	66.1 %	14.3 %	16.5 %	3.1 %	100 %
	Total	N	13370	2836	3383	654	20243
		% row	66.1 %	14.0 %	16.7 %	3.2 %	100 %

Supplemental Table S10.1. Descriptive statistics of the PA status; 1st, 5th, and 8th grade in the pre-pandemic and pandemic period.

		Pre-pandemic			During pandemic		
		Inactive	Irregularly active	Active	Inactive	Irregularly active	Active
1st	N	11119	64	3037	6324	3	496
	% row	78.2 %	0.5 %	21.3 %	92.7 %	0.04 %	7.26 %
	BMI $\overline{X}$	16.061	16.770	16.058	16.179	14.640	16.079
5th	N	6354	1124	13416	2600	64	2455
	% row	30.4 %	5.4 %	64.2 %	50.8 %	1.2 %	48.0 %
	BMI $\overline{X}$	19.276	19.170	18.961	19.440	19.332	19.130
8th	N	9417	1514	12746	4469	148	2620
	% row	39.8 %	6.4 %	53.8 %	61.8 %	2.0 %	36.2 %
	BMI $\overline{X}$	21.058	20.800	20.701	21.096	20.851	20.837

Arithmetic mean of body mass index (BMI $\overline{X}$)

Supplemental Table S10.2. Post hoc tests; Bonferroni tests; dependent variable: BMI; effect: PA status; 1st, 5th, and 8th grade in the pre-pandemic and pandemic period.

			Pre-pandemic		During pandemic	
			t	p_{bonf}	t	p_{bonf}
**1st**	Active	Inactive	- 0.066	1.000	- 0.915	1.000
		Irregular exercise	- 2.598	0.028	1.057	0.872
	Inactive	Irregular exercise	- 2.607	0.027	1.134	0.771
**5th**	Active	Inactive	- 6.087	< 0.001	- 3.059	0.007
		Irregular exercise	- 1.978	0.144	- 0.443	1.000
	Inactive	Irregular exercise	0.965	1.000	0.237	1.000
**8th**	Active	Inactive	- 7.574	< 0.001	- 2.880	0.012
		Irregular exercise	- 1.043	0.891	- 0.045	1.000
	Inactive	Irregular exercise	2.693	0.021	0.803	1.000

t-statistics (t) and Bonferroni statistical significance (p_{bonf})

Supplemental Table S10.3. Descriptive statistics of the PA status in relation to sex; 1st, 5th, and 8th grade

		Boys			Girls		
		Inactive	Irregularly active	Active	Inactive	Irregularly active	Active
1st	N	8886	37	1922	8557	30	1611
	% row	82.0 %	0.3 %	17.7 %	83.9 %	0.3 %	15.8 %
5th	N	4058	547	8560	4899	641	7334
	% row	30.8 %	4.2 %	65.0 %	38.0 %	5.0 %	57.0 %
8th	N	6104	734	8806	7779	928	6560
	% row	39.0 %	4.7 %	56.3 %	50.9 %	6.1 %	43.0 %
